# Genetic manipulation of the pigment pathway in a sea urchin reveals distinct lineage commitment prior to metamorphosis in the bilateral to radial body plan transition

**DOI:** 10.1038/s41598-020-58584-5

**Published:** 2020-02-06

**Authors:** Gary M. Wessel, Masato Kiyomoto, Tun-Li Shen, Mamiko Yajima

**Affiliations:** 1 Department of Molecular Biology, Cellular Biology, and Biochemistry, Brown University, Providence, RI 02912 USA; 2 Tateyama Marine Laboratory, Marine and Coastal Research Center, Ochanomizu University, Kou-yatsu 11, Tateyama, Chiba, 294-0301 Japan; 3 Department of Chemistry, Brown University, Providence, RI 02912 USA

**Keywords:** Biochemistry, Cell biology, Developmental biology, Evolution, Molecular biology, Zoology

## Abstract

Echinoderms display a vast array of pigmentation and patterning in larval and adult life stages. This coloration is thought to be important for immune defense and camouflage. However, neither the cellular nor molecular mechanism that regulates this complex coloration in the adult is known. Here we knocked out three different genes thought to be involved in the pigmentation pathway(s) of larvae and grew the embryos to adulthood. The genes tested were polyketide synthase (PKS), Flavin-dependent monooxygenase family 3 (FMO3) and glial cells missing (GCM). We found that disabling of the PKS gene at fertilization resulted in albinism throughout all life stages and throughout all cells and tissues of this animal, including the immune cells of the coelomocytes. We also learned that FMO3 is an essential modifier of the polyketide. FMO3 activity is essential for larval pigmentation, but in juveniles and adults, loss of FMO3 activity resulted in the animal becoming pastel purple. Linking the LC-MS analysis of this modified pigment to a naturally purple animal suggested a conserved echinochrome profile yielding a pastel purple. We interpret this result as FMO3 modifies the parent polyketide to contribute to the normal brown/green color of the animal, and that in its absence, other biochemical modifications are revealed, perhaps by other members of the large FMO family in this animal. The FMO modularity revealed here may be important in the evolutionary changes between species and for different immune challenges. We also learned that glial cells missing (GCM), a key transcription factor of the endomesoderm gene regulatory network of embryos in the sea urchin, is required for pigmentation throughout the life stages of this sea urchin, but surprisingly, is not essential for larval development, metamorphosis, or maintenance of adulthood. Mosaic knockout of either PKS or GCM revealed spatial lineage commitment in the transition from bilaterality of the larva to a pentaradial body plan of the adult. The cellular lineages identified by pigment presence or absence (wild-type or knock-out lineages, respectively) followed a strict oral/aboral profile. No circumferential segments were seen and instead we observed 10-fold symmetry in the segments of pigment expression. This suggests that the adult lineage commitments in the five outgrowths of the hydropore in the larva are early, complete, fixed, and each bilaterally symmetric. Overall, these results suggest that pigmentation of this animal is genetically determined and dependent on a population of pigment stem cells that are set-aside in a sub-region of each outgrowth of the pentaradial adult rudiment prior to metamorphosis. This study reveals the complex chemistry of pigment applicable to many organisms, and further, provides an insight into the key transitions from bilateral to pentaradial body plans unique to echinoderms.

## Introduction

Animal pigmentation is used for many different functions including sexual selection, species identification, protection from light, and camouflage. Pigmentation in echinoderms is complex and highly variable (see^1–3^ and the references therein). For example, a variety of carotenoids are prevalent in sea stars and are derived from a plant-based diet. Carotenoids are yellow, orange, or red fat-soluble terpenoid pigments, which give color to plant parts such as ripe tomatoes, autumn leaves, and is prevalent in many sea stars popular for research e.g. *Pateria miniata*. Melanins are synthesized by an animal through polymerization of oxidized tyrosine, but are sporadically found only in some echinoderms^4^. Porphyrins also are present sporadically in echinoderms but their origin in the animal is not yet clear. This family of pigments is characterized by a flat ring of four linked heterocyclic groups, sometimes with a central metal atom and includes heme and chlorophyll. Curiously, in echinoderms one species may have porphyrin pigment but a closely related species may not^5^. Their function in echinoderms is also not known^6^.

Quinones are oxidized aromatic compounds and one type of quinone, a naphthaquinone, is a major family of echinoderm pigmentation referred to as the echinochromes and spinochromes^7–10^. Historically, it was thought that only plants synthesized these quinone compounds until the echinochrome pigments were characterized first in sea urchins^3,11^. It is now clear though that animals are capable of synthesizing large amounts and varieties of naphthaquinone derivatives originating from a polymerization and cyclization of ketides. In sea urchins, this organic compound appears to be modified in a variety of ways; additions of aliphatic groups, hydroxylation, and/or oxidation^12^. Echinochrome pigments are thought to be involved in immune defense as an anti-microbial factor. Such activity by the pigment cells in larvae has been studied intensively, and appears to include pigment degranulation in the presence of microbial invaders, resulting in protection of the animal. Indeed, pigment isolated from the adult test and spines has been shown to have antimicrobial activity *in vitro*^13^.

The enzymatic pathway to make pigment in sea urchins involves a large, iterative-type polyketide synthase expressed in the larvae exclusively in the pigment cells^14^. PKS-null (Cas9-gRNA mediated genomic mutation) or knockdown (morpholino antisense oligonucleotide; MASO) larvae in two different species (*Strongylocentrotus purpuratus*, *Hemicentrotus pulcherrimus*) result in normal appearing development, but lacking in pigment^14–16^. A flavin-dependent monooxygenases (FMO3) also appears essential for larval pigmentation in *S*. *purpuratus*. Flavin-dependent monooxygenases comprise a large family of monoamine oxidases^17^ that are prevalent throughout phylogeny, although in most cases, their precise functions are not known. FMO3 was found in an mRNA-screen of pigment cells and when FMO3 is knocked down by a specific FMO3 MASO, the resulting larvae are also albino^14^. Analyzing the biochemical pathways and products for this pigment, it is postulated that PKS makes a polyketide (echinochrome/spinochrome) that is oxidized by one or another FMO that results in different colors or types of pigment chemistry. It should be noted that both the FMO3 and the PKS genes expressed in the larvae are exclusive to the pigment cells^14^.

The pigment cells of the sea urchin larvae are committed early in gastrulation and depend on the activity of the transcription factor, glial cells missing^18–20^. GCM is a transcription factor in veg2 cells involved in specification of non-skeletogenic mesoderm, including pigment cells, and is directly regulated by the Notch-Delta pathway of signaling as long as the GCM – positive cells are in contact with the Delta expressing cells. Following Delta depletion, GCM increases its expression by feedback co-dependent regulatory loops with the transcription factor Six1. Thus, the persistent GCM expression leads to fully-committed pigment cell specification at an early developmental time. As reported in *S*. *purpuratus*, GCM-null larvae are albino, yet all other features of the embryo, at least through development to 72 hours, otherwise appears normal^15^. Unpublished observations (Perillo, Spurrell, Oulhen *et al*.,) suggest that the pigment cells are still made in Gcm-null animals, but that these “pigment cells” lack pigment.

The biology of echinoderm pigment cells has largely focused on larval stages, which is essential for understanding the molecular details and the specification mechanisms of these specific cell types. However, it has been unclear if/how those pigment genes actually contribute to complex pigmentation patterning and immune defense in adulthood, both of which are essential for survivability. To understand the biological significance of pigmentation in the adults, we analyzed the consequences of pigment gene inactivation through larval life into adulthood using CRISPR/Cas9-mediated gene inactivation. Here, we report that all pigmentation of the sea urchin *H*. *pulcherrimus* requires a polyketide synthase and that FMO3 activity is required for modification of the pigment to a dark green – brown color normally prevalent in this adult. We use this pigmentation finding to study the body plan transition from bilaterally symmetric to pentaradial and learned that pigment stem cells follow strict oral-aboral boundaries in the pentaradial adult rudiment prior to metamorphosis. We also noticed that development, metamorphosis, and adult maintenance was independent of GCM and that the albino adult animals are less resistant to challenging environmental conditions compared to adult animals with pigment. This finding suggests a potential important role of these pigment genes in immune defense and other survivability mechanisms in the adult animals.

## Materials and Methods

### Animal culture

Eggs and sperm were collected from wild-type adults of *Hemicentrotus pulcherrimus* using 2 mM acetylcholine injection into the coelomic cavity of the adult. The embryos were cultured at 15 °C and the larvae were fed the diatom *Chaetoceros gracilis*, *ad libitum*. Metamorphosis was induced by adding pieces of plastic plate covered with calcareous red algae. The resulting juveniles were cultured at 15 °C and fed dried seaweed *Undaria pinnatifida ad libitum*.

### Cas9 mRNA/Guide RNAs (gRNAs) preparation

gRNAs were designed using CRISPRscan (www.crisprscan.org) to coding sequences of each gene at HpBase (http://cell-innovation.nig.ac.jp/Hpul/)^21^ and synthesized as reported^22^. The plasmid pCS2-3xFLAG-NLS-SpCas9-NLS was a gift from Yonglong Chen (Addgene plasmid #51307), and was linearized with NotI and transcribed with SP6. This transcript encodes Cas9 (codon optimized for mammalian cells) along with two nuclear localization sequences (NLS)^23^ and has been shown previously to be functional in sea urchin embryos (Shevidi *et al*., 2017). The gRNAs were synthesized by T7 RNA polymerase using the MegaShortScript T7 transcription kit (AM1354, ThermoFisher, Waltham, MA). The gRNAs were then purified using the miRNeasy Mini kit (217004, Qiagen, Valencia, CA).

### Microinjection and analysis of phenotypes

Two gRNAs (200 ng/ul of each gRNA) were mixed with 500 ng/ul of Cas9 mRNA, injected into freshly fertilized eggs as described previously in^24^. At 48 hrs, 72 hrs, and 5–10 days, the animals were photographed and measured. The larvae that showed no pigmentation between 5~10 days were selected and cultured to the adulthood. At one year of culture, the young adult animals were biopsied for coelomic fluids for pigment cell imaging (Olympus) and for genetic analysis.

### Identification of genomic mutations

Genomic DNA was isolated from several tube feet donated by each subject using 100 microliters of QuickExtract DNA Extraction Solution (http://www.epibio.com/) according to manufacturer’s instructions. One microliter of the extraction mix was then subjected to PCR amplification of the targeted genomic DNA region: 95 °C, 3 minutes, 95 °C, 15 seconds, 60 °C, 15 seconds, 72 °C, 30 seconds, 95 °C, 15 seconds, repeated 30 rounds. Primers to the genomic regions flanking the gRNA target site were used to amplify the locus (see Supplementary Table 2). Sequence of the PCR population was accomplished using the same amplification primers and mutation sites were identified by decomposition of trace chromatograms (https://tide.deskgen.com/)^25^; as well as individual clones of the gDNA amplicon.

### Pigment chemistry

Pigment in control and FMO3- animals was analyzed as reported^26^ by LC-MS with modifications. Briefly, spines were collected from adult animals, dried, crushed, macerated and then suspended in 1.0 ml of aqueous HCl 6 M for 1 h. The sample was then spun 1 minute at high speed in a microfuge and the supernatant was moved to a new tube. An equal volume of diethyl ether was added to the supernatant, and the tube was vortexed for 30 seconds and then spun at high speed in a microfuge. The diethyl ether fraction was moved to a new tube and partitioned against 1/10 volume aqueous NaCl 5% solution. The diethyl ether phase was removed and evaporated to dryness. The pigment extracts were resuspended in MeOH and were analyzed by liquid chromatography – mass spectrometry (LC-MS). Three main peaks were detected in the urchin extracts. The high resolution accurate MS data indicated the chemical formula of the main component correspond to major chromatographic peaks are Spinochrome A (C12H8O7), Spinochrome B (C10H6O6), and Spinochrome C (C12H8O8).

### LC-MS analysis

Sea urchin pigment analyses were performed using an HPLC system (1260 series, Agilent Technologies) coupled to a 6530 Accurate-Mass Q-TOF (Agilent Technologies) operated in negative (ESI-) electrospray ionization mode. Vials containing pigment were kept at −20 C prior to LC-MS analysis. Reversed phase column Waters XTerra MS C18, 3.5 μm 2.1 × 50 mm column was used at 40 C with a sample volume injected of 8 μL and flow rate of 0.3 μL/min. The HPLC mobile phases consist: A = 0.1% formic acid in water, B = acetonitrile. The linear gradient elution used the following time program: 0 min 5% B, linear to 95% B at 9.5 min, hold at 95% for 2 min, back to 5% B at 14 min, and equilibrate for 8 min. The injection volume was 8 μL. The ESI source conditions were gas temperature 300 C, drying gas 11 L/min, nebulizer 35 psig, VCap voltage 3500 V, fragmentor 175 V, and skimmer 65 V. The instrument was tuned using an Agilent calibration tuning mix for mass calibration of the Q-TOF instrument. The reference solution provided reference masses m/z 112.9856 and m/z 1033.9881 for ESI (−) were used to correct small mass drift during acquisition. Data were collected in both centroid and profile formates and data analysis used Agilent MassHunter Qualitative Analysis (v. B.06.00).

## Results

**Figure 1:**
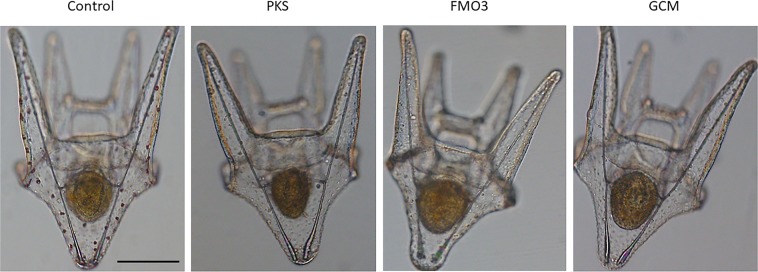
Images of larvae (*Hemicentrotus pulcherrimus*) are shown at 10 days post fertilization. Larvae were fed algae and can be seen with full stomachs. Wild-type control animals also have deep red pigment cells throughout the body of the larva. Animals in which guide RNAs were injected for PKS, FMO3, or GCM develop, swim, and feed normally, but they have no pigmentation. Scale bar = 100 microns.

We designed gRNAs to genomic DNA of the PKS (HPU_11477), GCM (HPU_07306), and FMO3 (HPU_04909) genes of *H*. *pulcherrimus*, targeting the N-terminal regions of the coding exons for maximal inactivation of gene function (Supplementary Table 2). Each gene inactivation resulted in albinism in the larvae, which served as a selectable marker for gene inactivation, but no other discernable differences in larval development or behavior were observed, even at 10 days post-fertilization (Fig. 1). In general, over 80% of the embryos injected with Cas9 mRNA and the gRNA pool resulted in albino larvae at 5–10 days post-fertilization, and only complete albino larvae were selected and subjected to further analysis in this study.

**Figure 2:**
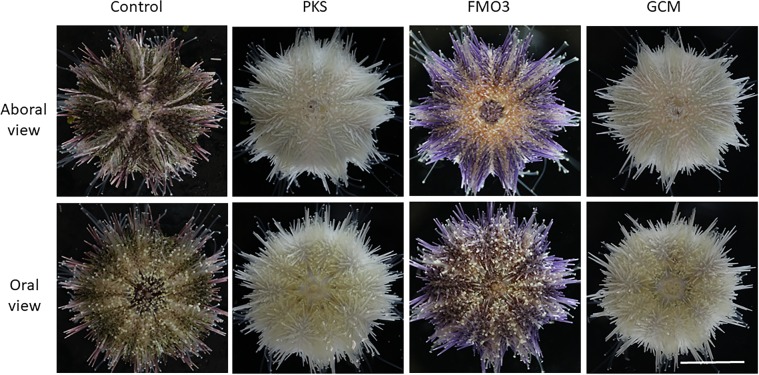
Aboral and oral views of animals used in this study (*Hemicentrotus pulcherrimus*) are shown. Animals lacking functional PKS gene are lacking pigment throughout the test, spines, and tube feet. The same phenotype is seen for animals lacking GCM. Animals lacking FMO3 instead reveal pastel purple throughout their spines, but all other features of the adult are the same as in the wild-type control. Scale bar = 1 cm.

**Figure 3:**
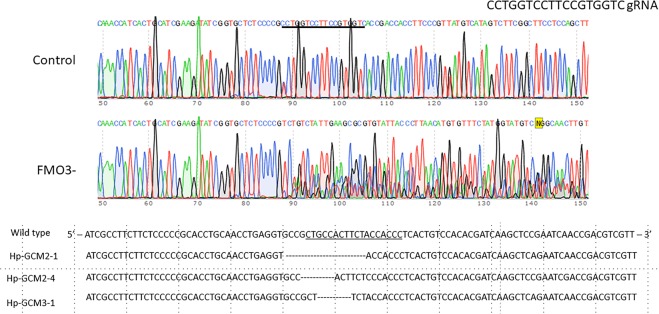
Genome analysis of mutations.

We attempted to grow PKS-, GCM-, and FMO3-null larvae from each of these sibling populations to metamorphosis and to adulthood (Fig. 2; Supplementary Table 1). Success in larval development, metamorphosis, and juvenile development to adults of wild-type animals (25%) was consistent with previous experiments on controls in which 20–40% of larvae reach adulthood on average. FMO3 success in reaching adulthood was somewhat greater at 37%, whereas PKS and GCM were slightly less (17% and 13% respectively). Further, juvenile growth rates are highly variable, even among siblings in both the wild-type and knock-out populations. Overall, none of the mutants appeared to have a significant deficit in larval development, metamorphosis, or juvenile development. Remarkably, even the GCM-null animals developed through larval stages^27^, metamorphosed into juveniles, and appeared as normal and healthy adults except that they had no pigment. The visual clue of albinism indicated that GCM had been mutated to a non-functional form, and is verified by genomic DNA analysis (Fig. 3), but it was surprising that this gene is non-essential for development of the key structures it appears to regulate in larvae. We conclude that other genes of the organism must compensate for the activity lost by GCM knockout, perhaps by a genetic compensation triggered by mutant mRNA degradation^28–30^. This conclusion is based on work in the closely related species, *Strongylocentrotus purpuratus*, where a large set of genes is identified to be regulated by GCM^18^, and by its key position within the gene regulatory network (http://www.echinobase.org/endomes/#Veg-21-30-NetworkDiagram). Currently, we do not have candidates in hand that may serve such compensatory roles. We note that for gcm and for the other genes tested in this study, we usually saw only 2 different genomic mutations in an animal, and never more than 3. We interpret this result as 2 or 3 independent “hits” by Cas9 that must have occurred within the first three cell divisions of embryogenesis. This timing is the same as reported earlier in larvae^15^ and argues that the gcm, pks, and fmo3 genes were inactivated prior to their normal timing of gene transcription.

**Figure 4:**
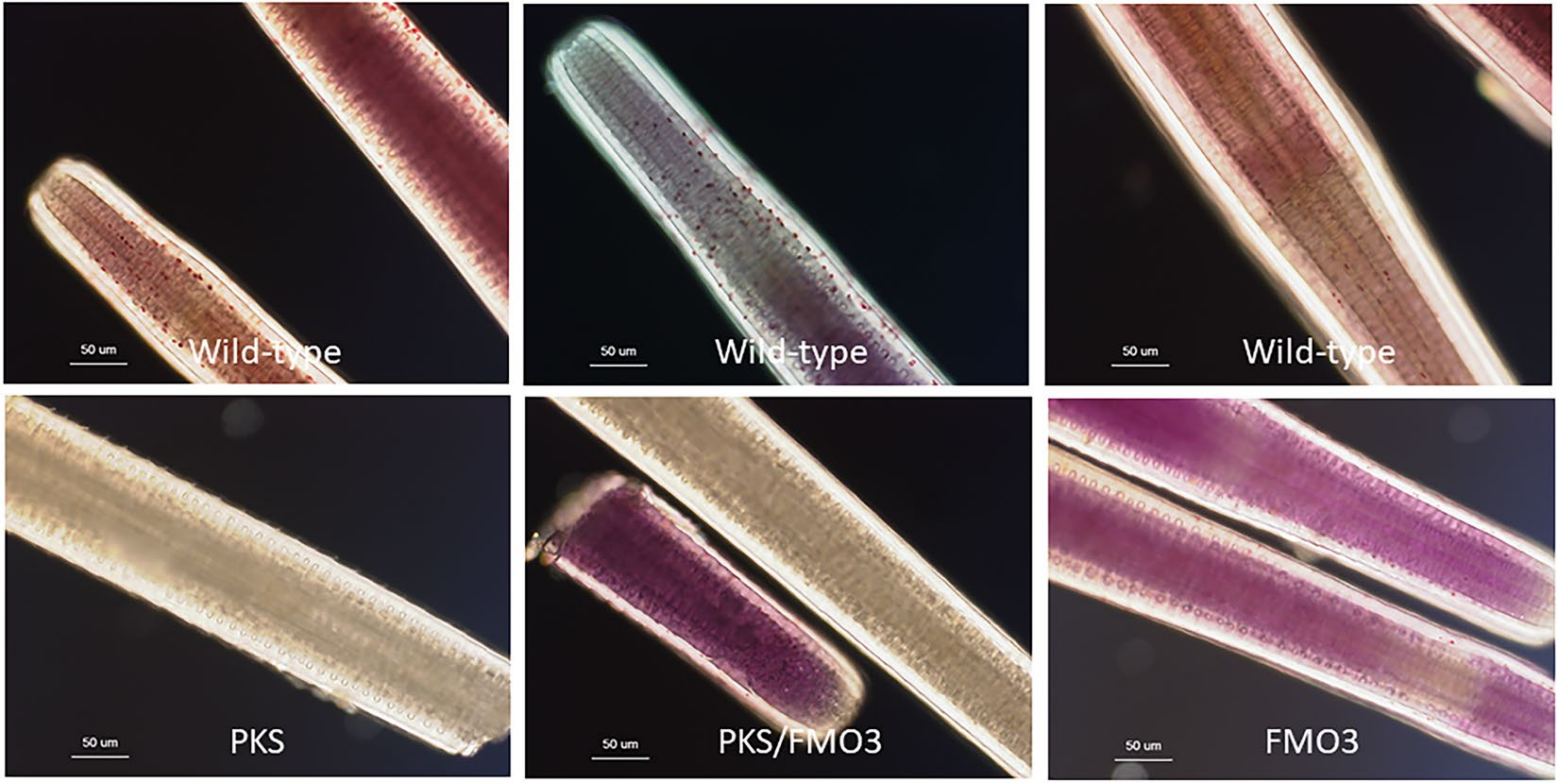
Close up of spines from wild-type, PKS- and FMO3-null animals. Wild-type spines have two sources of pigment, that in the individual cells throughout the surface (stroma) of the spine, and that throughout the complex latticework (stereom) of the spine. Note the variation in spine color in the wild-type spines (from one individual) and the cross-sectional patches of more or less pigment in the core of the spine. PKS-null spines have neither pigment in individual cells at the surface, nor in the core of the spine. FMO3 spines on the other hand have a pastel purple throughout the core of the spine and an occasional single cell of pigment at the surface. The overall morphology of the spines in each case is otherwise indistinguishable from each other, and GCM has the same phenotype as does PKS (data not shown). Scale bar = 50 microns.

**Figure 5:**
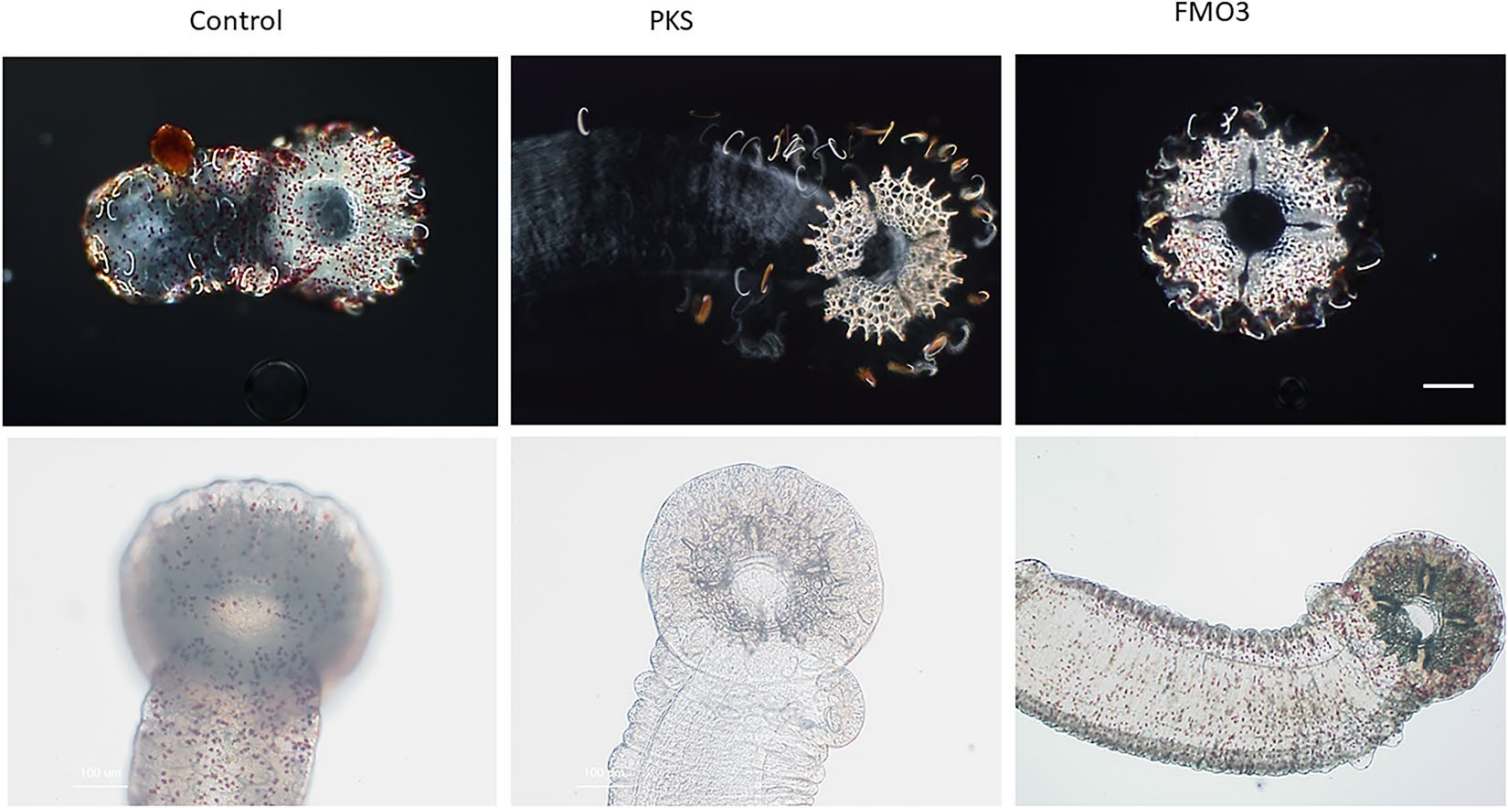
Tube feet are shown under polarized and DIC microscopy. Tube feet from control animals show the detailed patterning of the skeletal elements characteristic of this tissue as well as the intense red pigment of the pigment cells throughout the tip and base of each tube foot. The tube feet from animals lacking PKS also contain intricate skeletal elements in their tube feet, but have no detectable pigment in the tip, base, or elsewhere in the tube feet. This phenotype is indistinguishable from the GCM-null animals (data not shown). Tube feet of the FMO3-null animals appear indistinguishable from the wild-type tube feet. They have detailed skeletal elements, and pigment cells, seen in the tips of the tube feet by polarized light, and in the base of the tube feet seen by DIC. Scale bar = 50 microns.

The PKS-null animals and the GCM-null animals were indistinguishable (Fig. 2). Both of these knock-out animal populations developed on schedule with controls animals, metamorphosed with similar frequency as the controls, and the juveniles and adults were otherwise normal. We note that all of the pigment of this animal appears dependent upon a PKS product. This includes pigment of the spines, test, tube feet, and even the tips of the tube feet, where the photoreceptors are enriched (Figs. 4 and 5). Further, upon biopsy of the coelomic fluid of these animals, we discovered the multiple classes of coelomocytes characteristically present. Yet, none of these cells had any detectable pigment in strong contrast to the normal enrichment (Fig. 4). Thus, we conclude that all pigment in this animal is derived from a polyketide based precursor.

Cas9 targeting of FMO3 resulted in the same phenotype of albinism in larvae (Fig. 1) and these larvae were screened and selected based on lack of larval pigment. When these animals metamorphosed and grew to adulthood, however, they resulted in distinct pigmentation. Instead of the dark green pigmentation consistent with control sibling *H*. *pulcherrimus* adults cultured in the same water table and fed the same diet, the FMO3-KO animals were a pastel purple color throughout most of the spines of the animal (Fig. 2). All other aspects of the adult, however, were otherwise indistinguishable from controls. This altered pigment was largely only on the spines of the adult, whereas the test, the coelomocytes, the tube feet and even the tips of the tube feet where the light sensitive opsins exist contained the characteristic dark red/brown pigment of the wild-type animals (Figs. 4 and 5). This FMO3-KO animal provided a unique opportunity to understand some of the biochemical mechanism for pigment synthesis and modification in this animal. While the PKS-null (and GCM-null) adults were stark white, indicative of the calcium carbonate skeleton of the spicules and test with a complete loss of pigment, the FMO3-null animals instead had a distinct change in pigment. FMO3 is here proposed to modify (oxidize) the base polyketide made by PKS to contribute to the dark green pigmentation that is characteristic of *H*. *pulcherrimus* and lack of its activity in the adult revealed other PKS-derivatives, perhaps modified differently by other FMO-family members. Other FMOs and other types of modification enzymes may also be functional in this pigment biosynthesis. Since the larvae were initially screened for albinism, and the FMO3-KO larvae were albino, we conclude that FMO3 is essential for modification in pigmentation of the larvae, whereas in the juvenile and adult, other FMO enzymes and/or polyketide modifiers are also present to create the FMO3-KO/pastel purple pigment.

**Figure 6:**
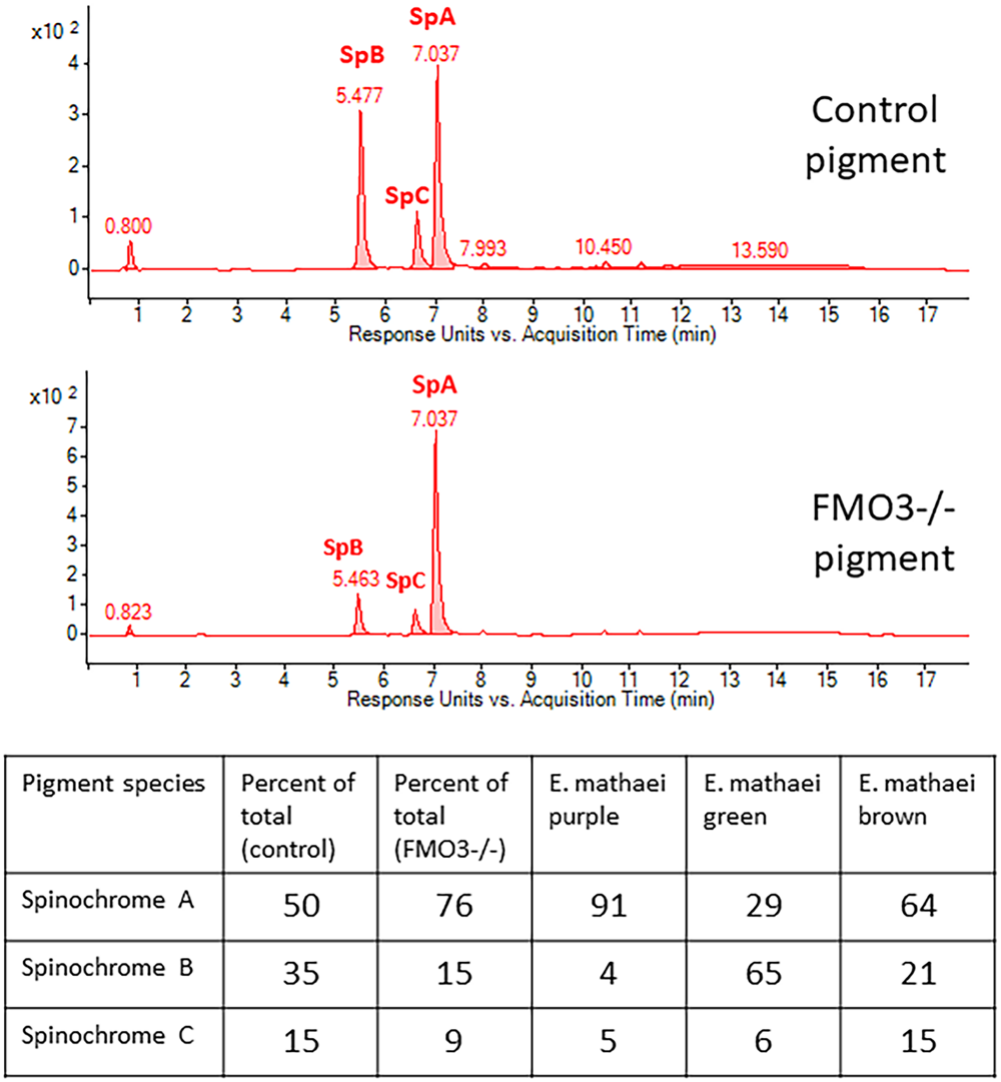
Pigment chemistry. LC-MS analysis of pigment extracted from spines of control and FMO3-null animals reveal that the ratios of each pigment expressed in spines changes dramatically when FMO3 is absent. The pigment changes and resulting purple color are consistent with the purple variant in a recent study of diversity of pigments in a different sea urchins, *E*. *mateai*.

To test if FMO3-null animals indeed have a different pigment composition, their spines were analyzed and compared to controls by LC-MS (Fig. 6). It should be noted that the control animals have a diversity of spine colors, including red/purple/green/brown (Fig. 2) whereas the FMO3-null animals have predominantly purple spines. Thus, our analysis was an attempt to determine what pigment FMO3 was responsible for in its biosynthesis within a background of varied colors in the control. The results showed that the control spines had three major pigment peaks that corresponded to Spinochromes A, B and C. The order of abundance of the pigments in wild-type animals was A, B, and C in a ratio of 5:3.5:1.5 and quite consistent between three separate extractions. Other lesser pigments were also present but not pursued in this analysis. The FMO3−/− animals showed the same three major peaks but the ratios of the pigment was distinctly different from the wild-type animals (7.5:1.5:1). Thus, we conclude that FMO3 contributes to the accumulation of Spinochrome B, but we are unable to determine currently if Spinochrome A is modified to become Spinochrome B, or if the pathway of Spinochrome B biosynthesis is independent. Of note is a recent study of pigment in *Echinometra mathaei*, a species with at least four distinct colored spines. Brasseur *et al*.^26^, characterize the composition of pigments in each color variant, and identified the pigment ratios, the palate of pigments responsible for each color in this animal. Importantly, the ratios of each pigment in the purple variant were similar to those of the purple color of the FMO3-null *H*. *pulcherrimus* animals, compared to the green or brown variants. Thus, we propose that FMO3 modifies Spinochrome A to yield Spinochrome B and C.

**Figure 7:**
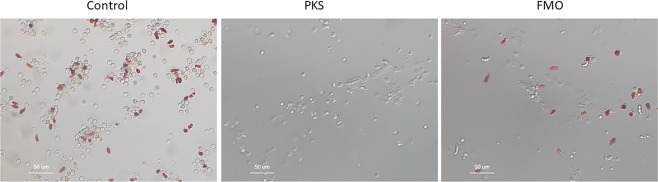
Coelomocytes were biopsied from adults and imaged by DIC. Wild-type animals have diverse cells types, include the intensely red-pigmented red spherule cells. This diverse population of coelomocytes and pigment cells is also present in the FMO3-null animals. PKS-null animals also have diverse coelomocytes but no pigment is present. Scale bar = 50 microns.

Coelomocytes are the adult immune cells and they permeate the body cavity of the adult. The sea urchin harbors several different types of coelomocytes with presumed different functions. Some coelomocytes are pigmented and degranulate these contents upon a microbial infection. Upon inspection of PKS – KO animals, the several various types of coelomocytes were observed, but no pigment was present in any of these cells. As best we could tell, the “red” spheroidal cells were still present, but they lacked pigment (Fig. 7). When coelomocytes from FMO3-null animals were analyzed, they appeared indistinguishable from those of control animals both in terms of amount of pigment, and color of pigment. We conclude from this analysis that FMO3 is not required for pigmentation in the pigmented coelomocytes, but that the coelomocyte pigment is polyketide based and is synthesized by PKS.

It should be noted that a previous test of PKS-null animals resulted in the same success of larval development, metamorphosis and anatomical normality, but several months after metamorphosis, all of the PKS-KO animals (47 total) died within 2 weeks of each other (Supplemental Fig. 6). The sibling controls of these PKS-KOs were harbored in the same water tables and they all (over 100) remained healthy. Only the albino animals died. Upon dissection of these animals, no morphological abnormalities were seen (data not shown). Because of the link between echinochrome pigment and immune defense^31^, we surmise that the albino animals succumbed to a microbe(s) for which pigmented animals (wild-type and other targeted gene Cas9-gRNA animals) were otherwise immune.

**Figure 8:**
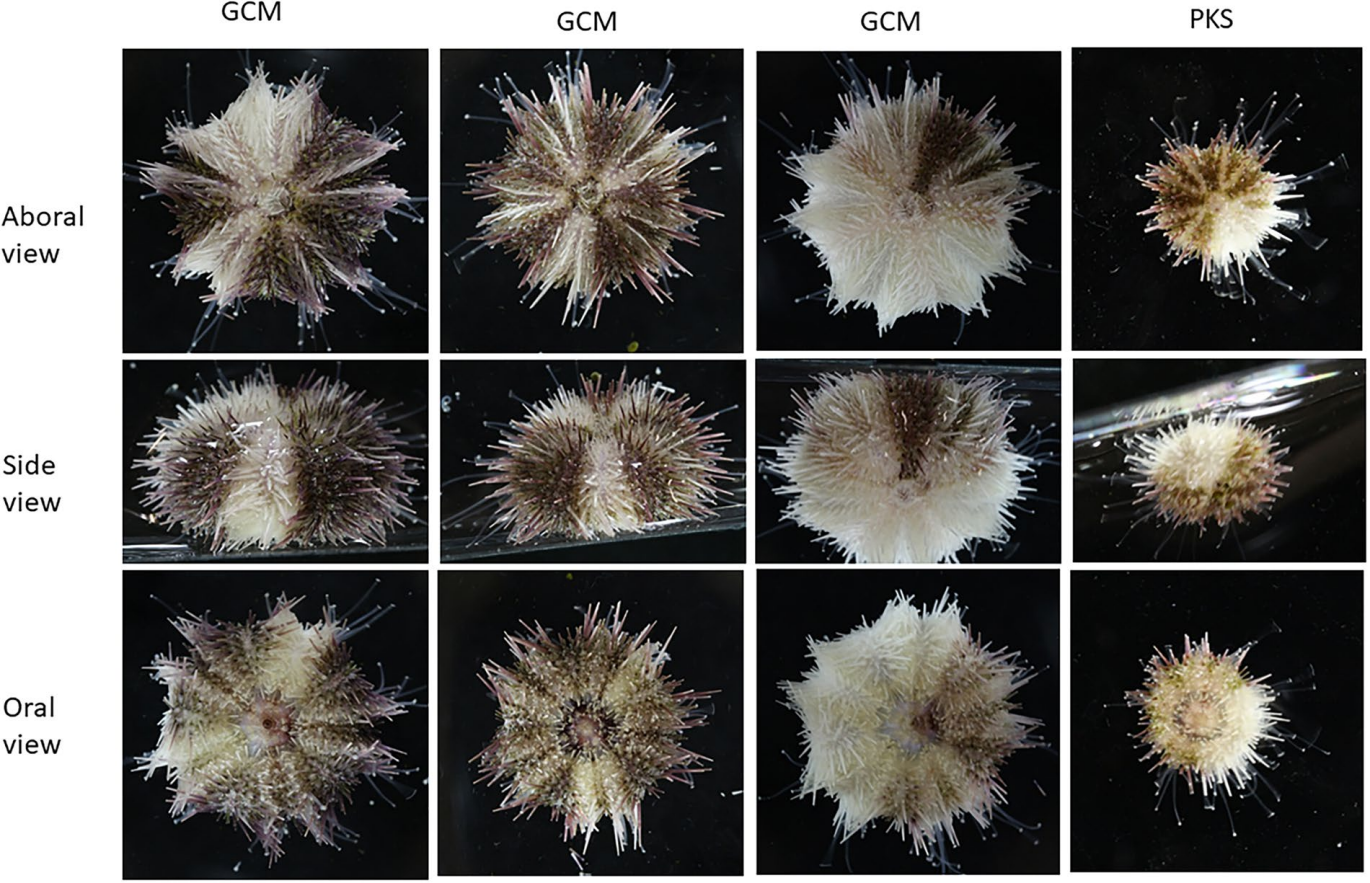
Mosaic pigmentation was seen in PKS- and GCM-null animals. Shown are aboral, side, and oral views of representatives of the mosaics. Note that in segments lacking pigmentation, all external structures are lacking pigment, including the test, spines, and tube feet and that these regions extend longitudinally, from oral to aboral.

Mosaicism of pigmentation was seen in several of the GCM-KO and PKS-KO animals (Fig. 8). We interpret this result to indicate that the Cas9/gRNA mutation was not complete and may have resulted in mutated cells adjacent to non-mutated cells in the early embryo. What is striking about these mosaic animals is how tight the boundary is between regions of pigmentation and albinism. Radiality (adult body plan) forms in bilaterally symmetric larvae as a consequence of the pentameric outgrowths of the hydropore, a circumpharyngeal tube that forms in early larvae^32,33^. How the positions of the pentameric outgrowth are regulated is not clear, but these mosaic animals suggest that placement of pigment cells, or the stem cell line responsible for pigmentation, is not random, nor is there significant mixing of these cells during the elaborations of each out-pocketing. The pattern of the mosaicism is also striking in that boundaries are seen only in an oral/aboral direction and not in a circumferential pattern that crosses the 5 bilaterally symmetric plates of the adult. Indeed, some mosaic animals are seen where half of the animal is non-pigmented (5/10), or 1/10^th^ of the animal is pigmented or not. These patterns suggest a population of pigment stem cells is finely placed within each boundary of a hydropore out-pocketing prior to metamorphosis, resulting in a consistent oral/aboral pattern throughout metamorphosis and adult development.

## Discussion

Pigment has been an important tool for understanding developmental mechanisms. Naturally-pigmented cells enabled classical investigators to follow tissue changes and morphological shifts over time even at a subcellular level. Perhaps most famously, the grey crescent in *Triturus* enabled Mangold and Spemann^34^ to identify the organizer, a morphologically active zone for instructing later development. The interpretations of their subsequent transplant experiments were definitive because they were able to leverage a natural pigment marker. Here we use Cas9-KOs to probe how pigment is formed in a sea urchin, and as a lineage mapping mechanism to explore how the transition from bilateral to radial symmetry of the body plan might be managed.

**Figure 9:**
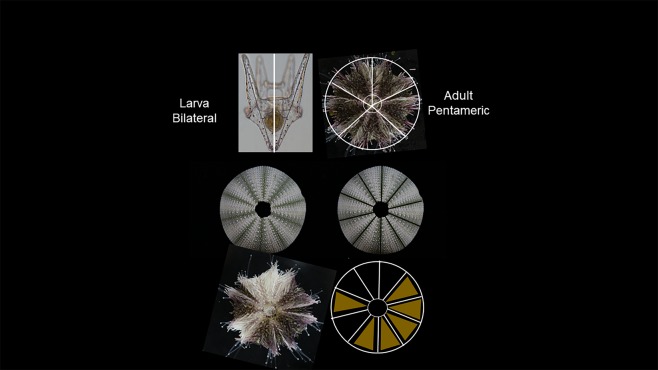
Model of pigmentation mosaics in the transition from bilaterality to a radial body plan. A bilaterally symmetric larva adjacent to a test of *H*. *pulcherrimus* with spines and tube feet removed that reveals a major 5-fold symmetry characteristic of this phyla and a minor division of these of the five sections to yield 10 repeating segments. Each of the pentameric regions contain a bilateral region that may have different pigmentation. Pigmentation in each of the mosaics follows a 10-fold symmetry (5 times bilateral symmetry, enabling for example, half-pigmented animals, or a small slice (1/10) of albinism, or both, as present in this animal.

The mosaic animals obtained in this study were derived, we surmise, by Cas9-mediated genomic mutation in only some cells of the early embryo. Since the larvae were selected visually by lack of pigmentation we conclude that the mutation(s) occurred within the Veg2 cells that give rise to the larval pigment cells, but not completely in the cells that give rise to adult pigment. In the event that a few pigments cells were missed when screening the larvae, such that they could contribute to pigment in the adult, we would have anticipated that the missed pigment cells would have more broadly migrated and proliferated throughout the adult such that discreet boundaries would be less likely to form in the adult. We extend this interpretation to conclude that the adult pigment cells arise from a different lineage from the larval pigment cells. This lineage appears to be parsed out into 10 separable oral-aboral bounded lineages. This conclusion is based on the observation that in the pigmented mosaic animals we analyzed, each of the boundaries and territories fit into a derivative of 10. The animal in e.g. Fig. 8, has two separated pigment territories, one of which is exactly ½ of the animal (5/10) and one of which is 1/10 of the adult body. Consistenly, the other mosaics too follow this 10-fold territory separation of the adult body plan (Fig. 9). At least in the adult test, the developmental lineages appear to follow a decameric oral-aboral symmetry. Mosaic animals were seen in both the PKS-KO and the GCM-KO, so we conclude these pigmentation chimerisms are not a gene-specific phenomenon.

*H*. *pulcherrimus* has 22 FMO/FMO-like genes in its genome^21^. In species in which these enzyme functions have been studied, the FMO family has wide ranging activities, including detoxification of metabolites and environmental purturbants^17^. In the closely related sea urchin species, *S*. *purpuratus*, we know five FMOs are enriched in the larval pigment cells, for which at least FMO3 also appears essential for larval pigment formation. The pigment of larval pigment cells in *H*. *pulcherrimus* is usually a deep red color and consistent between individuals even though the pigmentation of the adults may be quite distinct and variable. Indeed, *L*. *variegatus* larvae have a deep red pigment in their pigment cells of the larva but the adult spines, and test range from white, to green, to red and to many colors in-between. Thus, we anticipate that the base polyketide of PKS is essential in other sea urchins as well, and that variations in FMO expression and/or activities will drive the variations in the adult tissues. Given such a broad enzymatic family as FMO, coupled with a conserved base compound of the echinochrome polyketide resulting from PKS, it may not be surprising then that variations in the expression of different members of the FMO family that act on the polyketide would result in significant variations in the palate of colors seen in the adult. It is noteworthy that given the promiscuity of FMO activity that the FMO3-KO only resulted in phenotypic changes in color, and only apparent in the spines of the adult. No differences were seen in development, morphogenesis, adult structures, or behavior. We conclude that a main function of FMO3 is in modifying a base polyketide into the dark green/brown pigment characteristic of *H*. *pulcherrimus* and in its absence, the polyketide modified by yet other FMO family members are more apparent as a pastel purple phenotype. Individuals of the species of sea urchin used in this study, *Hemicentrotus pulcherrimus*, are relatively homogeneous in pigmentation.

The result of this study suggests that PKS and its echinochrome base polyketide is essential for all pigmentation in the animal, at all stages in the life cycle. We surmise that what differs amongst different life stages, especially larva and adult pigment cells, are different combinations of FMO functionality in time and space. Such differences may also be in play for differences between species. We anticipate that the genetically modified animals here will have great utility in studying cell lineages of embryos and adults. The genetic modification is traceable for blastomere transplant experiments, and for manipulations leading to adult development, the pigment markers are an indelible, and are easy visual markers for endpoint metrics.

## Supplementary information

.Supplemental Figure 1

Supplemental Figure 2

Supplemental Figure 3

Supplemental Figure 4

Supplemental Figure 5

Supplemental Figure 6.

Supplementary Table 1

Supplementary Table 2
