# Supplementary figures and images for: Genetic manipulation of the pigment pathway in a sea urchin reveals distinct lineage commitment prior to metamorphosis in the bilateral to radial body plan transition
Source: Sci Rep. 2020 Feb 6;10:1973. doi: 10.1038/s41598-020-58584-5 (PMC7005274; doi:10.1038/s41598-020-58584-5)

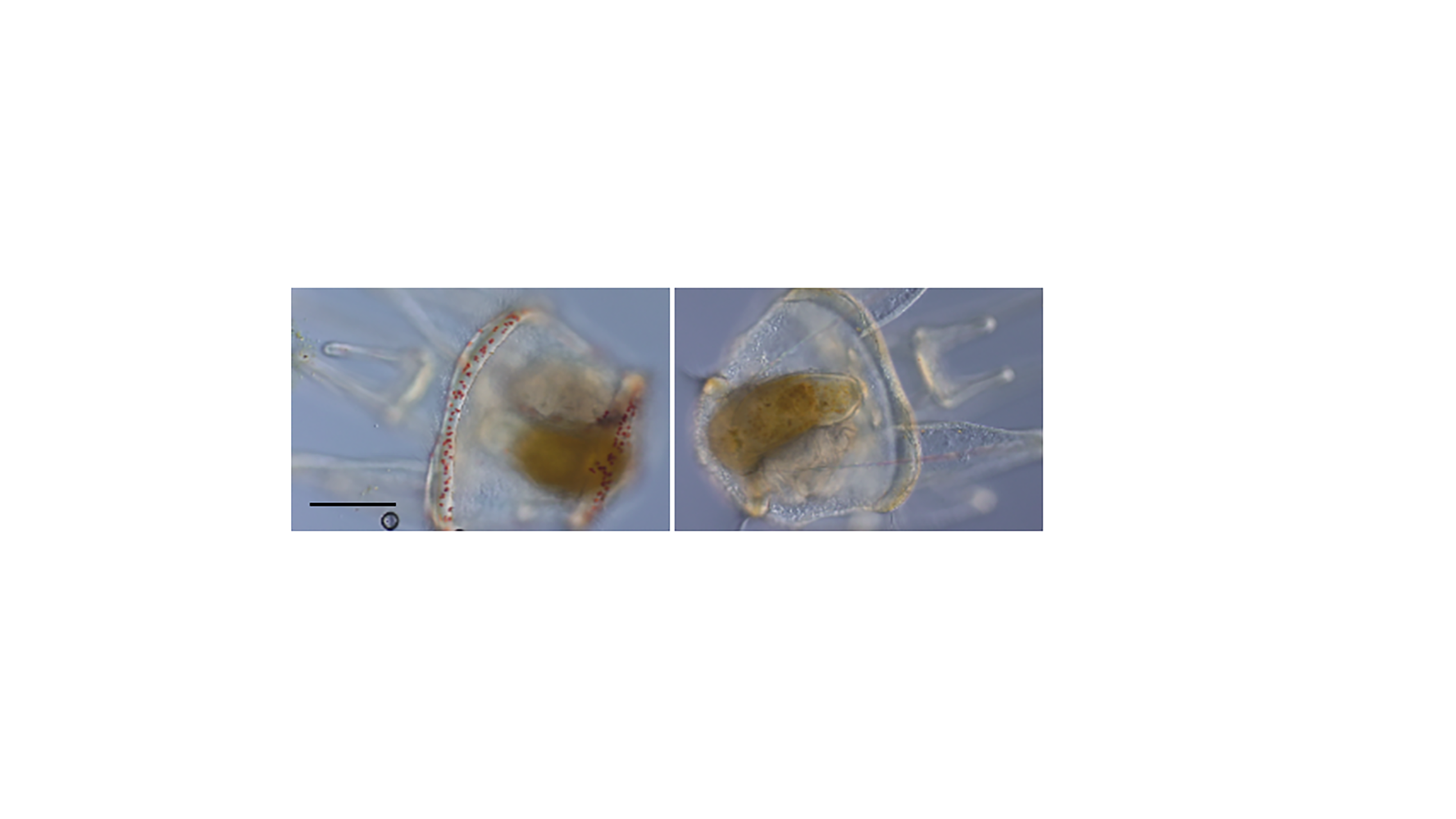

Supplement: Supplementary file 1 — .Supplemental Figure 1 [file 41598_2020_58584_MOESM1_ESM.tif]

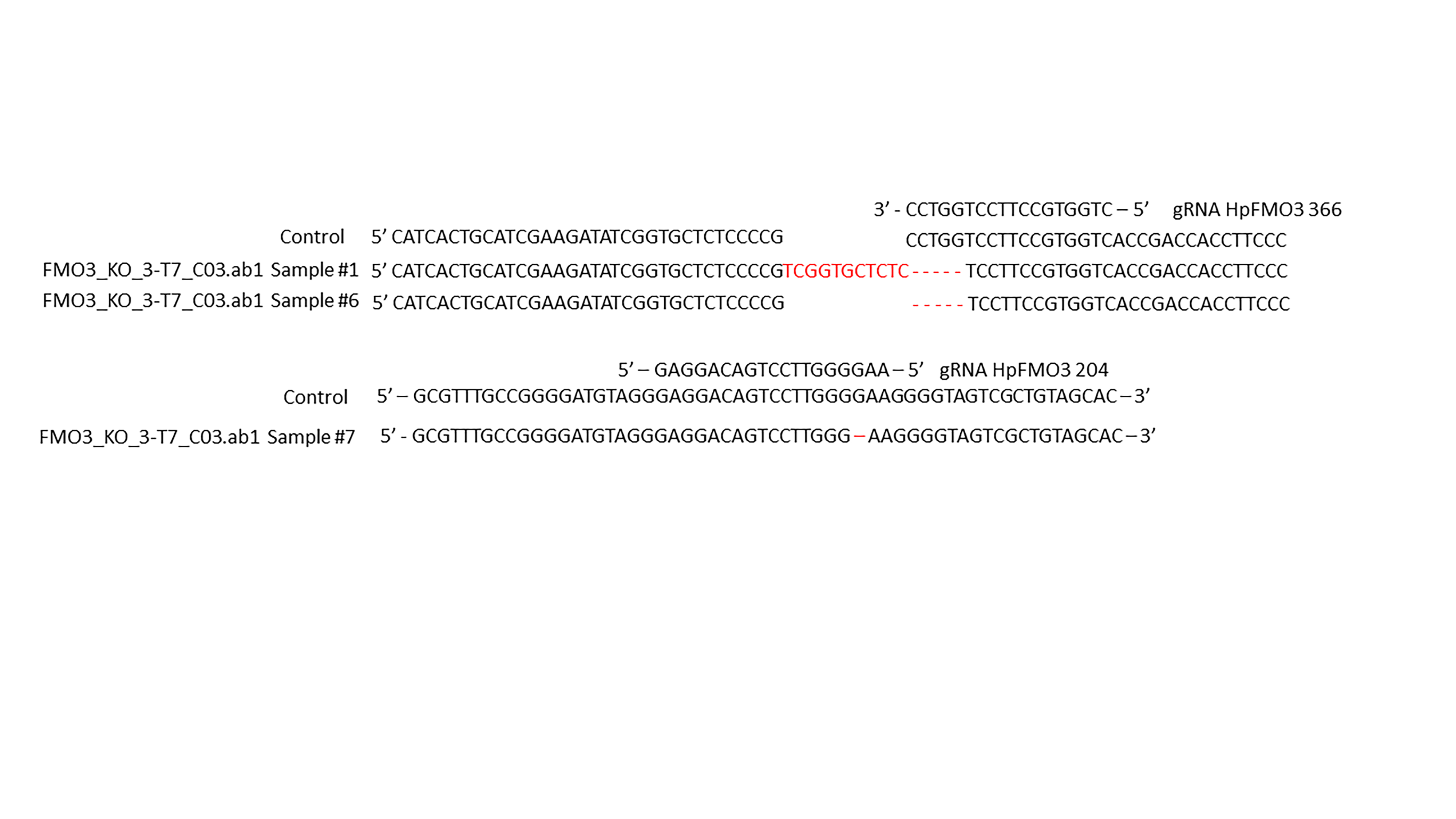

Supplement: Supplementary file 2 — Supplemental Figure 2 [file 41598_2020_58584_MOESM2_ESM.tif]

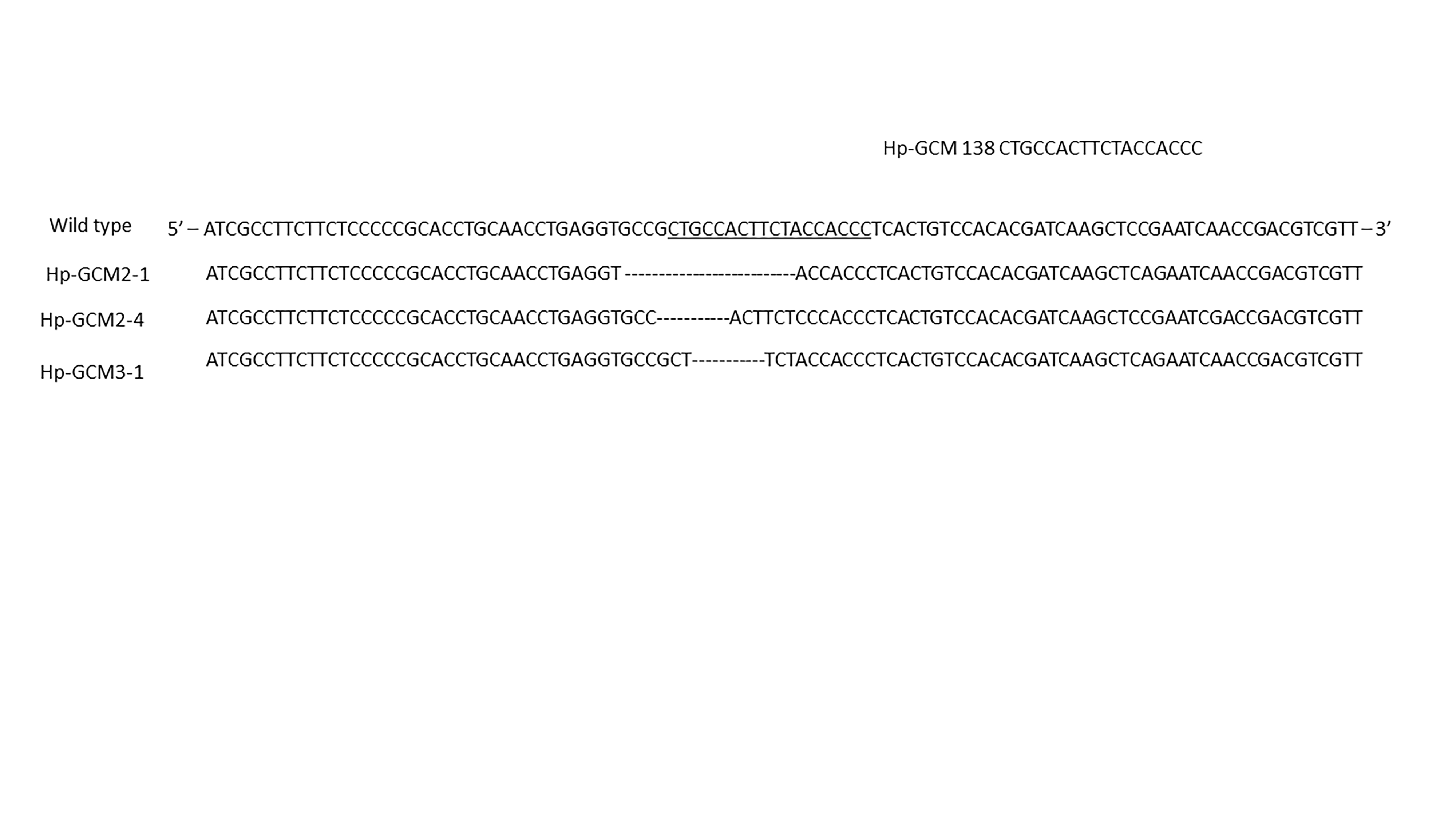

Supplement: Supplementary file 3 — Supplemental Figure 3 [file 41598_2020_58584_MOESM3_ESM.tif]

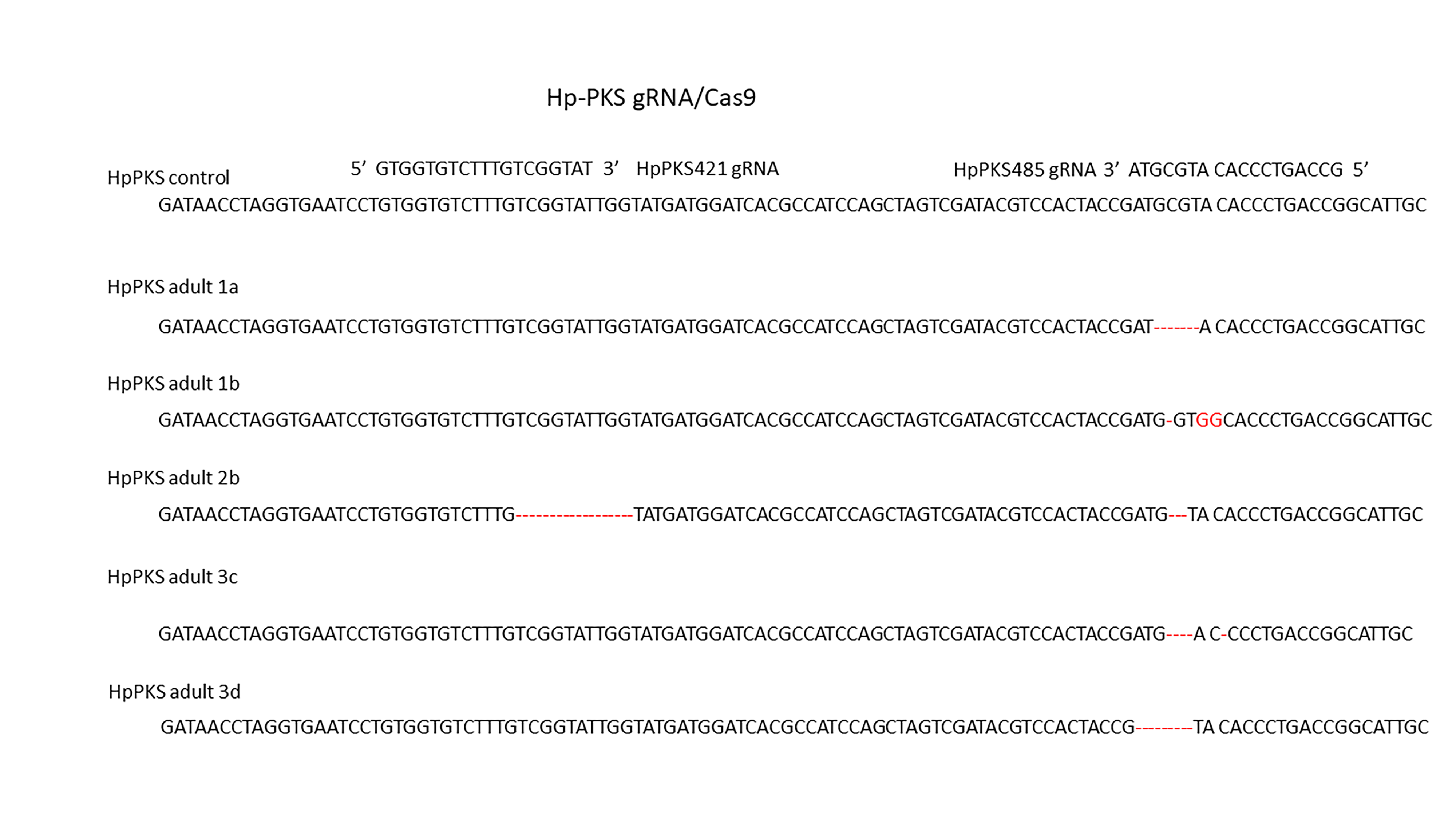

Supplement: Supplementary file 4 — Supplemental Figure 4 [file 41598_2020_58584_MOESM4_ESM.tif]

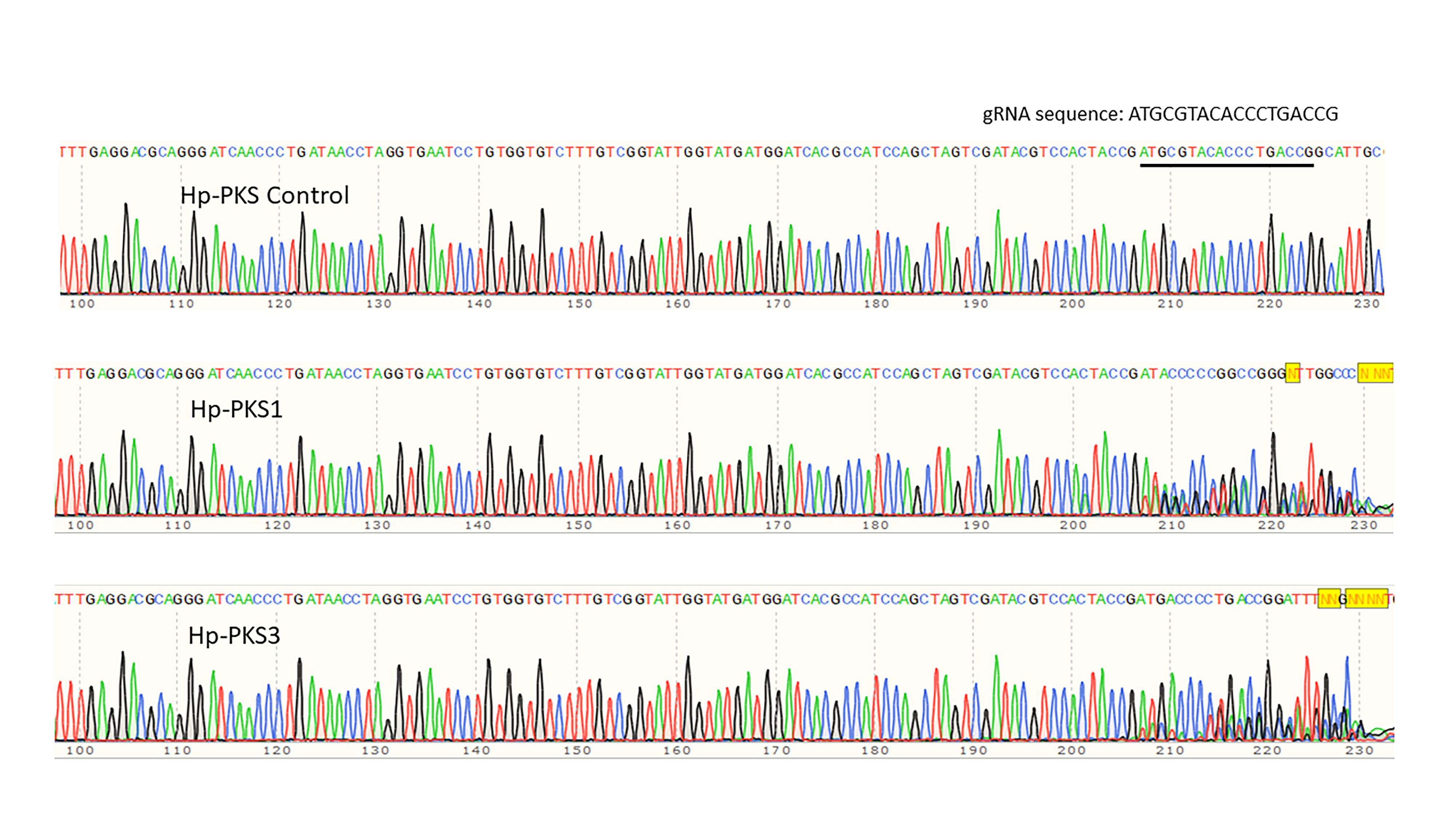

Supplement: Supplementary file 5 — Supplemental Figure 5 [file 41598_2020_58584_MOESM5_ESM.tif]

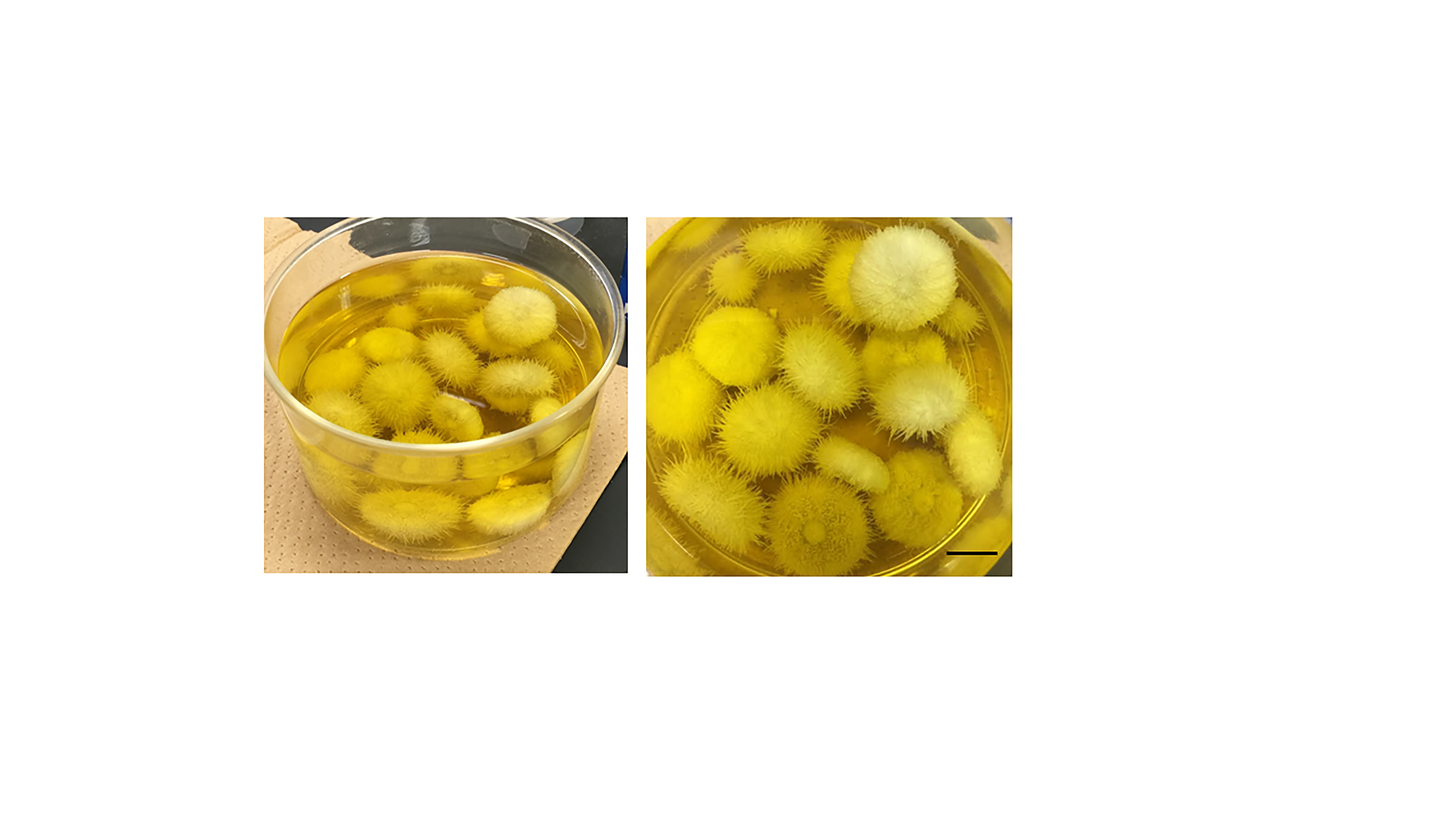

Supplement: Supplementary file 6 — Supplemental Figure 6. [file 41598_2020_58584_MOESM6_ESM.tif]
