# Supplementary material for: Genetic manipulation of the pigment pathway in a sea urchin reveals distinct lineage commitment prior to metamorphosis in the bilateral to radial body plan transition
Source: Sci Rep. 2020 Feb 6;10:1973. doi: 10.1038/s41598-020-58584-5 (PMC7005274; doi:10.1038/s41598-020-58584-5)
Supplement: Supplementary file 7 — Supplementary Table 1 [file 41598_2020_58584_MOESM7_ESM.docx]

Supplementary Table 1

Developmental progression of wild-type, PKS-null, Fmo3-null, and Gcm-null animals

Since each gene impacts pigment production, our selection was made visually, and the lack of pigment indicated gene inactivation.

| Gene manipulation/developmental time (post-fertilization) | Wild-type | Polyketide synthase (PKS) | Flavin containing monoxygenase (Fmo) 3 | Glial Cells Missing (GCM) |
| --- | --- | --- | --- | --- |
| Day 3 (larva; after selection for albinism in knock-out animals) | 600 | 270 | 255 | 195 |
| 4 weeks | 470 (78%) | 157 (58%) | 183 (72%) | 182 (93%) |
| 6.5 weeks (fully grown 8-armed larvae) | 441 (74%) | 148 (55%) | 173 (68%) | 35 (18%) |
| 30 weeks (juvenile) | 203 (34%) | 100 (37%) | 112 (44%) | 25 (13%) |
| 45 weeks | 147 (25%) | 45 (17%; 1 mosaic) | 95 (37%) | 25 (13%; 9 albino, 14 pigmented, 3 mosaic) |

Normal animals were uninjected. Percentiles were calculated based on number of day 3 or day 5 larvae selected. Over 80% of animals injected with GCM, PKS, or FMO3 gRNA/Cas9 were albino at the 3- or 5-day selection.
