# Supplementary material for: Genetic manipulation of the pigment pathway in a sea urchin reveals distinct lineage commitment prior to metamorphosis in the bilateral to radial body plan transition
Source: Sci Rep. 2020 Feb 6;10:1973. doi: 10.1038/s41598-020-58584-5 (PMC7005274; doi:10.1038/s41598-020-58584-5)
Supplement: Supplementary file 8 — Supplementary Table 2 [file 41598_2020_58584_MOESM8_ESM.docx]

Supplementary Table 2: gRNAs and primers to amplify target loci

| Gene | gRNA | Sequence (5’-3’) |
| --- | --- | --- |
| Polyketide synthase (PKS) HPU_11477 |  |  |
|  | HpPKS 82-105 | GGAGCAATAAAACCAGCTGG |
|  | HpPKS 101-123 | GGGGAACTCGCCATGCTTG |
|  | HpPKS 134-157 | GGGAACCTTCCAGAAGTCAT |
|  | HpPKS 297-320 | GGGGCGAGATCTTGAAGAAC |
|  | HpPKS 421-444 | GGTGGTGTCTTTGTCGGTAT |
|  | HpPKS 485-508 | GGCGGTCAGGGTGTACGCAT |
|  |  |  |
| Flavin-containing monoxygenase 3 (Fmo3)  HPU_04909 |  |  |
|  | HpFMO3 26-49 | GGGAGCTGGTGTGAGTGGCT |
|  | HpFMO3 98-121 | GGGGGACGAGAAAGTTGACG |
|  | HpFMO3 152-175 | GGGAGTTGGTAATCAGGGCG |
|  | HpFMO3 204-227 | GGAGGACAGTCCTTGGGGAA |
|  | HpFMO3 333-356 | GGCGAAGACTATGACATCAC |
|  | HpFMO3 366-389 | GGGACCACGGAAGGACCAGG |
|  |  |  |
| Glial cells missing (GCM)  HPU_07306 |  |  |
|  | Hp GCM 23-46 | GGGCGTGGTTATCAGTCGCC |
|  | Hp GCM 75-98 | GGAGCCTCGGATTCAGCAAC |
|  | Hp GCM 138-161 | GGGGGTGGTAGAAGTGGCAG |
|  | Hp GCM 263-286 | GGACGAGGTCGAGAGTTTGG |
|  | Hp GCM 302-325 | GGCCGCCGGAGCTGCCGGAT |
|  | Hp GCM 351-374 | GGGCAGGAGAAGGGAACTAG |
|  |  |  |
